# Supplementary material for: Can Extensive Training Transform a Mouse into a Guinea Pig? An Evaluation Based on the Discriminative Abilities of Inferior Colliculus Neurons
Source: Biology (Basel). 2024 Feb 2;13(2):92. doi: 10.3390/biology13020092 (PMC10886615; doi:10.3390/biology13020092)
Supplement: Supplementary file 1 [file biology-13-00092-s001.zip › biology-2781531-supplementary.pdf]

Table S1 （Original data of **Figure 3**）

Variable	Mean value	SEM	n (value)	P**	F (DFn. DFd)	R square	Units	Statistical test	Figure
Firing rate all groups	Trained: 29.509<br>Exposed: 23.545<br>Guinea Pig: 23.379	Trained: 2.566<br>Exposed: 1.578<br>Guinea Pig: 1.150	Trained: 316<br>Exposed: 268<br>Guinea Pig:390	0.0218*	F (2. 971) = 3.843	0.007852	Spike/second	ANOVA	3.A
Corrcoeff all groups	Trained: 0.458<br>Exposed: 0.396<br>Guinea Pig:0.397	Trained: 0.016<br>Exposed: 0.014<br>Guinea Pig:0.012	Trained: 303<br>Exposed: 264<br>Guinea Pig:359	0.0009 ***	F (2. 923) = 7.082	0.01511	none	ANOVA	3.B
MI all groups	Trained: 1.204<br>Exposed: 1.106<br>Guinea Pig:2.011	Trained: 0.055<br>Exposed: 0.059<br>Guinea Pig:0.031	Trained: 74<br>Exposed: 59<br>Guinea Pig:342	0.0061**	F (2. 472) = 5.149	0.02135	bit	ANOVA	3.C

Variable	Mean value	SEM	n (value)	P**	T	DF	η^2 (eta squared)	Units	Statistical test	Figure in which data are presented
Firing rate Trained vs Exposed mice	Trained: 29.51<br>Exposed: 23.55	Trained: 2.57<br>Exposed: 1.52	Trained: 316<br>Exposed: 268	0.0519	1.948	582	0.006477	Spike/second	Unpaired T.test	3.A
Firing rate Trained VS guinea Pig	Trained: 29.51<br>Guinea Pig: 23.38	Trained: 2.57<br>Guinea Pig: 1.15	Trained: 316<br>Guinea Pig:389	0.0184 *	2.363	704	0.007868	Spike/second	Unpaired T.test	3.A
Firing rate Exposed VS guinea Pig	Exposed: 23.55<br>Guinea Pig: 23.38	Exposed: 1.52<br>Guinea Pig: 1.15	Exposed: 268<br>Guinea Pig:389	0.9294	0.08862	656	1.197e-005	Spike/second	Unpaired T.test	3.A

Variable	Mean value	SEM	n (value)	P**	T	DF	η^2 (eta squared)	Units	Statistical test	Figure in which data are presented
Corrcoeff<br>Trained vs Exposed mice	Trained: 0.458<br>Exposed: 0.396	Trained: 0.016<br>Exposed: 0.014	Trained: 303<br>Exposed: 264	0.0033 **	2.950	565	0.01516	none	Unpaired T.test	3.B
Corrcoeff Trained VS guinea Pig	Trained: 0.458<br>Guinea Pig:0.397	Trained: 0.016<br>Guinea Pig:0.012	Trained: 303<br>Guinea Pig:359	0.0008 ***	3.355	660	0.01677	none	Unpaired T.test	3.B
Corrcoeff Exposed VS guinea Pig	Exposed: 0.396<br>Guinea Pig:0.397	Exposed: 0.014<br>Guinea Pig:0.012	Exposed: 264<br>Guinea Pig:359	0.9465	0.06712	621	7.255e-006	none	Unpaired T.test	3.B
MI Trained vs Exposed mice	Trained: 1.204<br>Exposed: 1.106	Trained: 0.055<br>Exposed: 0.059	Trained: 74<br>Exposed: 59	0.2282	1.211	131	0.01106	bit	Unpaired T.test	3.C
MI Trained VS guinea Pig	Trained: 1.204<br>Guinea Pig:1.011	Trained: 0.055<br>Guinea Pig:0.031	Trained: 74<br>Guinea Pig:342	0.0024 **	3.056	414	0.02207	bit	Unpaired T.test	3.C
MI Exposed VS guinea Pig	Exposed: 1.106<br>Guinea Pig:1.011	Exposed: 0.059<br>Guinea Pig:0.031	Exposed: 59<br>Guinea Pig:342	0.1719	1.369	399	0.004673	bit	Unpaired T.test	3.C

Table S2 (Original data of **Figure 4A**)

Variable	Mean value	SEM	n (value)	P**	F (DFn. DFd)	R square	Units	Statistical test	Figure in which data are presented
Firing rate all groups<br>SNR +10	Trained: 18.111<br>Exposed: 15.224<br>Guinea Pig:20.687	Trained: 1.751<br>Exposed: 1.233<br>Guinea Pig:1.032	Trained: 316<br>Exposed: 268<br>Guinea Pig:390	0.0166 *	F (2. 971) = 4.113	0.008401	Spike/second	ANOVA	4.A
Firing rate all groups<br>SNR +0	Trained: 15.335<br>Exposed: 13.808<br>Guinea Pig:17.306	Trained: 1.699<br>Exposed: 1.261<br>Guinea Pig:0.947	Trained: 316<br>Exposed: 268<br>Guinea Pig:390	0.1564	F (2. 971) = 1.859	0.003815	Spike/second	ANOVA	4.A
Firing rate all groups<br>SNR -10	Trained: 16.773<br>Exposed: 12.422<br>Guinea Pig: 16.973	Trained: 1.990<br>Exposed: 0.900<br>Guinea Pig:1.080	Trained: 316<br>Exposed: 268<br>Guinea Pig:390	0.0466 *	F (2. 971) = 3.077	0.006297	Spike/second	ANOVA	4.A

Variable	Mean value	SEM	n (value)	P**	T	DF	η^2 (eta squared)	Units	Statistical test	Figure in which data are presented
Trained vs Exposed<br>SNR+10	Trained: 18.111<br>Exposed: 15.224	Trained: 1.751<br>Exposed: 1.233	Trained: 316<br>Exposed: 268	0.1858	1.325	582	0.003006	Spike/second	Unpaired T.test	4.A
Trained VS guinea Pig SNR+10	Trained: 18.111<br>Guinea Pig:20.687	Trained: 1.751<br>Guinea Pig:1.032	Trained: 316<br>Guinea Pig:390	0.1806	1.340	704	0.002545	Spike/second	Unpaired T.test	4.A
Exposed VS guinea Pig SNR+10	Exposed: 15.224<br>Guinea Pig:20.687	Exposed: 1.233<br>Guinea Pig:1.032	Exposed: 268<br>Guinea Pig:390	0.0007 ***	3.391	656	0.01723	Spike/second	Unpaired T.test	4.A

Variable	Mean value	SEM	n (value)	P**	T	DF	η^2 (eta squared)	Units	Statistical test	Figure in which data are presented
Trained vs Exposed SNR +0	Trained: 15.335<br>Exposed: 13.808	Trained: 1.699<br>Exposed: 1.261	Trained: 316<br>Exposed: 268	0.4768	0.7119	582	0.0008700	Spike/second	Unpaired T.test	4.A
Trained VS guinea Pig SNR +0	Trained: 15.335<br>Guinea Pig:17.306	Trained: 1.699<br>Guinea Pig:0.947	Trained: 316<br>Guinea Pig:390	0.2811	1.079	704	0.001650	Spike/second	Unpaired T.test	4.A
Exposed VS guinea Pig SNR +0	Exposed: 13.808<br>Guinea Pig:17.306	Exposed: 1.261<br>Guinea Pig:0.947	Exposed: 268<br>Guinea Pig:390	0.0242 *	2.259	656	0.007718	Spike/second	Unpaired T.test	4.A
Trained vs Exposed<br>SNR -10	Trained: 16.773<br>Exposed: 12.422	Trained: 1.990<br>Exposed: 0.900	Trained: 316<br>Exposed: 268	0.0556	1.918	582	0.006283	Spike/second	Unpaired T.test	4.A
Trained VS guinea Pig<br>SNR -10	Trained: 16.773<br>Guinea Pig: 16.973	Trained: 1.990<br>Guinea Pig:1.080	Trained: 316<br>Guinea Pig:390	0.925	0.09414	704	1.259e-005	Spike/second	Unpaired T.test	4.A
Exposed VS guinea Pig<br>SNR -10	Exposed: 12.422<br>Guinea Pig: 16.973	Exposed: 0.900<br>Guinea Pig:1.080	Exposed: 268<br>Guinea Pig:390	0.0025 **	3.030	656	0.01380	Spike/second	Unpaired T.test	4.A

Table S3 （Original data of **Figure 4B**）

Variable	Mean value	SEM	n (value)	P**	F (DFn. DFd)	R square	Units	Statistical test	Figure in which data are presented
Corrcoeff<br>all groups<br>SNR +10	Trained: 0.289<br>Exposed: 0.253<br>Guinea Pig:0.369	Trained: 0.017<br>Exposed: 0.015<br>Guinea Pig:0.012	Trained: 293<br>Exposed: 259<br>Guinea Pig:338	<0.0001<br>****	F (2. 887) = 17.95	0.03889	none	ANOVA	4.B
Corrcoeff<br>all groups<br>SNR +0	Trained: 0.217<br>Exposed: 0.183<br>Guinea Pig:0.263	Trained: 0.016<br>Exposed: 0.015<br>Guinea Pig:0.010	Trained: 275<br>Exposed: 258<br>Guinea Pig:342	<0.0001<br>****	F (2. 872) = 9.640	0.02163	none	ANOVA	4.B
Corrcoeff<br>all groups<br>SNR -10	Trained: 0.108<br>Exposed: 0.076<br>Guinea Pig:0.121	Trained: 0.011<br>Exposed: 0.009<br>Guinea Pig:0.006	Trained: 275<br>Exposed: 259<br>Guinea Pig:344	0.0009 ***	F (2. 875) = 7.047	0.01585	none	ANOVA	4.B

**Variable**	**Mean value**	**SEM**	**n (value)**	**P****	**T**	**DF**	**η^2 (eta squared)**	**Units**	**Statistical test**	**Figure in which data are presented**
Trained vs Exposed<br>SNR+10	Trained: 0.289<br>Exposed: 0.253	Trained: 0.017<br>Exposed: 0.015	Trained: 293<br>Exposed: 259	0.1153	1.577	550	0.004504	none	Unpaired T.test	4.B
Trained VS guinea Pig SNR+10	Trained: 0.289<br>Guinea Pig:0.369	Trained: 0.017<br>Guinea Pig:0.012	Trained: 293<br>Guinea Pig:338	<0.0001<br>****	4.118	629	0.02625	none	Unpaired T.test	4.B
Exposed VS guinea Pig<br>SNR+10	Exposed: 0.253<br>Guinea Pig:0.369	Exposed: 0.015<br>Guinea Pig:0.012	Exposed: 259<br>Guinea Pig:338	<0.0001<br>****	6.249	595	0.06159	none	Unpaired T.test	4.B

Variable	Mean value	SEM	n (value)	P**	T	DF	η^2 (eta squared)	Units	Statistical test	Figure in which data are presented
Trained vs Exposed SNR +0	Trained: 0.217<br>Exposed: 0.183	Trained: 0.016<br>Exposed: 0.015	Trained: 275<br>Exposed: 258	0.1125	1.590	531	0.004736	none	Unpaired T.test	4.B
Trained VS guinea Pig SNR +0	Trained: 0.217<br>Guinea Pig:0.263	Trained: 0.016<br>Guinea Pig:0.010	Trained: 275<br>Guinea Pig:342	0.0097 **	2.594	615	0.01082	none	Unpaired T.test	4.B
Exposed VS guinea Pig SNR +0	Exposed: 0.183<br>Guinea Pig:0.263	Exposed: 0.015<br>Guinea Pig:0.010	Exposed: 258<br>Guinea Pig:342	<0.0001 ****	4.685	598	0.03541	none	Unpaired T.test	4.B
Trained vs Exposed SNR -10	Trained: 0.108<br>Exposed: 0.076	Trained: 0.011<br>Exposed: 0.009	Trained: 275<br>Exposed: 259	0.0226 *	2.286	532	0.009731	none	Unpaired T.test	4.B
Trained VS guinea Pig SNR -10	Trained: 0.108<br>Guinea Pig:0.121	Trained: 0.011<br>Guinea Pig:0.006	Trained: 275<br>Guinea Pig:344	0.2952	1.048	617	0.001776	none	Unpaired T.test	4.B
Exposed VS guinea Pig SNR -10	Exposed: 0.076<br>Guinea Pig:0.121	Exposed: 0.009<br>Guinea Pig:0.006	Exposed: 259<br>Guinea Pig:344	<0.0001 ****	4.225	601	0.02885	none	Unpaired T.test	4.B

Table S4 （Original data of **Figure 4C**）

Variable	Mean value	SEM	n (value)	P**	F (DFn. DFd)	R square	Units	Statistical test	Figure in which data are presented
MI<br>all groups<br>SNR +10	Trained: 0.942<br>Exposed:0.817<br>Guinea Pig:1.018	Trained: 0.069<br>Exposed: 0.065<br>Guinea Pig:0.034	Trained: 56<br>Exposed: 48<br>Guinea Pig:389	0.0220 *	F (2. 409) = 3.850	0.01848	bit	ANOVA	4.C
MI<br>all groups<br>SNR +0	Trained: 0.872<br>Exposed: 0.763<br>Guinea Pig:0.863	Trained: 0.073<br>Exposed: 0.069<br>Guinea Pig:0.033	Trained: 46<br>Exposed: 40<br>Guinea Pig:389	0.3946	F (2. 358) = 0.9322	0.005181	bit	ANOVA	4.C
MI<br>all groups<br>SNR -10	Trained: 0.757<br>Exposed: 0.692<br>Guinea Pig:0.621	Trained: 0.089<br>Exposed: 0.068<br>Guinea Pig:0.027	Trained: 28<br>Exposed: 25<br>Guinea Pig:389	0.1868	F (2. 227) = 1.690	0.01467	bit	ANOVA	4.C

Variable	Mean value	SEM	n (value)	P**	T	DF	η^2 (eta squared)	Units	Statistical test	Figure in which data are presented
Trained vs Exposed SNR+10	Trained: 0.942<br>Exposed:0.817	Trained: 0.069<br>Exposed: 0.065	Trained: 56<br>Exposed: 48	0.1983	1.295	102	0.01617	bit	Unpaired T.test	4.C
Trained VS guinea Pig SNR+10	Trained: 0.942<br>Guinea Pig:1.018	Trained: 0.069<br>Guinea Pig:0.034	Trained: 56<br>Guinea Pig:389	0.2811	1.080	362	0.003209	bit	Unpaired T.test	4.C
Exposed VS guinea Pig SNR+10	Exposed:0.817<br>Guinea Pig:1.018	Exposed: 0.065<br>Guinea Pig:0.034	Exposed: 48<br>Guinea Pig:389	0.0069 **	2.717	354	0.02042	bit	Unpaired T.test	4.C

Variable	Mean value	SEM	n (value)	P**	T	DF	η^2 (eta squared)	Units	Statistical test	Figure in which data are presented
Trained vs Exposed SNR +0	Trained: 0.872<br>Exposed: 0.763	Trained: 0.073<br>Exposed: 0.069	Trained: 46<br>Exposed: 40	0.2865	1.073	84	0.01351	bit	Unpaired T.test	4.C
Trained VS guinea Pig SNR +0	Trained: 0.872<br>Guinea Pig:0.863	Trained: 0.073<br>Guinea Pig:0.033	Trained: 46<br>Guinea Pig:389	0.9037	0.1211	319	4.598e-005	bit	Unpaired T.test	4.C
Exposed VS guinea Pig SNR +0	Exposed: 0.763<br>Guinea Pig:0.863	Exposed: 0.069<br>Guinea Pig:0.033	Exposed: 40<br>Guinea Pig:389	0.1765	1.355	313	0.005828	bit	Unpaired T.test	4.C
Trained vs Exposed SNR -10	Trained: 0.757<br>Exposed: 0.692	Trained: 0.089<br>Exposed: 0.068	Trained: 28<br>Exposed: 25	0.5726	0.818	50	0.001586	bit	Unpaired T.test	4.C
Trained VS guinea Pig SNR -10	Trained: 0.757<br>Guinea Pig:0.621	Trained: 0.089<br>Guinea Pig:0.027	Trained: 28<br>Guinea Pig:389	0.0318*	1.632	203	0.01296	bit	Unpaired T.test	4.C
Exposed VS guinea Pig SNR -10	Exposed: 0.692<br>Guinea Pig:0.621	Exposed: 0.068<br>Guinea Pig:0.027	Exposed: 25<br>Guinea Pig:389	0.2429	1.171	201	0.006778	bit	Unpaired T.test	4.C

Table S5 （Original data of Figure 5A）

Variable	Mean value	SEM	n (value)	P**	F (DFn. DFd)	R square	Units	Statistical test	Figure in which data are presented
Firing rate all groups SNR +10	Trained:23.122 Exposed:13.279 Guinea Pig:20.010	Trained:2.013 Exposed:1.401 Guinea Pig:1.030	Trained:316 Exposed:268 Guinea Pig:390	0.163	F (2. 971) = 1.817	0.003730	Spike/second	ANOVA	5.A
Firing rate all groups SNR +0	Trained:25.076 Exposed:20.312 Guinea Pig:19.886	Trained:2.116 Exposed:1.445 Guinea Pig:1.043	Trained:316 Exposed:268 Guinea Pig:390	0.0293 *	F (2. 971) = 3.543	0.007245	Spike/second	ANOVA	5.A
Firing rate all groups SNR -10	Trained:25.190 Exposed:20.154 Guinea Pig:24.871	Trained:2.077 Exposed:1.301 Guinea Pig:1.295	Trained:316 Exposed:268 Guinea Pig:390	0.0587	F (2. 971) = 2.844	0.005825	Spike/second	ANOVA	5.A

Variable	Mean value	SEM	n (value)	P**	T	DF	η^2 (eta squared)	Units	Statistical test	Figure in which data are presented
Trained vs Exposed SNR+10	Trained:23.122 Exposed:13.279	Trained:2.013 Exposed:1.401	Trained:316 Exposed:268	0.1238	1.541	582	0.004064	Spike/second	Unpaired T.test	5.A
Trained VS guinea Pig SNR+10	Trained:23.122 Guinea Pig:20.010	Trained:2.013 Guinea Pig:1.030	Trained:316 Guinea Pig:390	0.1399	1.478	704	0.003092	Spike/second	Unpaired T.test	5.A
Exposed VS guinea Pig SNR+10	Exposed:13.279 Guinea Pig:20.010	Exposed:1.401 Guinea Pig:1.030	Exposed:268 Guinea Pig:390	0.6678	0.4293	656	0.0002809	Spike/second	Unpaired T.test	5.A

Variable	Mean value	SEM	n (value)	P**	T	DF	η^2 (eta squared)	Units	Statistical test	Figure in which data are presented
Trained vs Exposed SNR +0	Trained:25.076<br>Exposed:20.312	Trained:2.116<br>Exposed:1.445	Trained:316<br>Exposed:268	0.0685	1.825	582	0.005689	Spike/second	Unpaired T.test	5.A
Trained VS guinea Pig SNR +0	Trained:25.076<br>Guinea Pig:19.886	Trained:2.116<br>Guinea Pig:1.043	Trained:316<br>Guinea Pig:390	0.0182 *	2.368	704	0.007899	Spike/second	Unpaired T.test	5.A
Exposed VS guinea Pig SNR +0	Exposed:20.312<br>Guinea Pig:19.886	Exposed:1.445<br>Guinea Pig:1.043	Exposed:268<br>Guinea Pig:390	0.8061	0.2455	656	9.190e-005	Spike/second	Unpaired T.test	5.A
Trained vs Exposed SNR -10	Trained:25.190<br>Exposed:20.154	Trained:2.077<br>Exposed:1.301	Trained:316<br>Exposed:268	0.0453 *	1.541	582	0.006869	Spike/second	Unpaired T.test	5.A
Trained VS guinea Pig SNR -10	Trained:25.190<br>Guinea Pig:24.871	Trained:2.077<br>Guinea Pig:1.295	Trained:316<br>Guinea Pig:390	0.8908	1.478	704	2.677e-005	Spike/second	Unpaired T.test	5.A
Exposed VS guinea Pig SNR -10	Exposed:20.154<br>Guinea Pig:24.871	Exposed:1.301<br>Guinea Pig:1.295	Exposed:268<br>Guinea Pig:390	0.0132 *	0.4293	656	0.009326	Spike/second	Unpaired T.test	5.A

Table S6（Original data of **Figure 5B**）

Variable	Mean value	SEM	n (value)	P**	F (DFn. DFd)	R square	Units	Statistical test	Figure in which data are presented
Corrcoeff<br>all groups<br>SNR +10	Trained:0.372<br>Exposed:0.332<br>Guinea Pig:0.363	Trained:0.015<br>Exposed:.0.014<br>Guinea Pig:0.012	Trained:305<br>Exposed:264<br>Guinea Pig:348	0.0912	F (2. 914) = 2.400	0.005225	none	ANOVA	5.B
Corrcoeff<br>all groups<br>SNR +0	Trained:0.348<br>Exposed:0.318<br>Guinea Pig:0.321	Trained:0.016<br>Exposed:0.014<br>Guinea Pig:0.012	Trained:304<br>Exposed:266<br>Guinea Pig:351	0.2263	F (2. 918) = 1.488	0.003232	none	ANOVA	5.B
Corrcoeff<br>all groups<br>SNR -10	Trained:0.354<br>Exposed:0.324<br>Guinea Pig:0.313	Trained:0.017<br>Exposed:.0.016<br>Guinea Pig:0.011	Trained:309<br>Exposed:261<br>Guinea Pig:365	0.0987	F (2. 932) = 2.321	0.004957	none	ANOVA	5.B

Variable	Mean value	SEM	n (value)	P**	T	DF	η^2 (eta squared)	Units	Statistical test	Figure in which data are presented
Trained vs Exposed<br>SNR+10	Trained:0.372<br>Exposed:0.332	Trained:0.015<br>Exposed:.0.014	Trained:305<br>Exposed:264	0.0477 *	1.984	567	0.006898	none	Unpaired T.test	5.B
Trained VS guinea Pig SNR+10	Trained:0.372<br>Guinea Pig:0.363	Trained:0.015<br>Guinea Pig:0.012	Trained:305<br>Guinea Pig:348	0.6283	0.4843	651	0.0003602	none	Unpaired T.test	5.B
Exposed VS guinea Pig<br>SNR+10	Exposed:0.332<br>Guinea Pig:0.363	Exposed:.0.014<br>Guinea Pig:0.012	Exposed:264<br>Guinea Pig:348	0.0689	1.822	610	0.005415	none	Unpaired T.test	5.B

Variable	Mean value	SEM	n (value)	P**	T	DF	η^2 (eta squared)	Units	Statistical test	Figure in which data are presented
Trained vs Exposed SNR +0	Trained:0.348 Exposed:0.318	Trained:0.016 Exposed:0.014	Trained:304 Exposed:266	0.1512	1.437	568	0.003624	none	Unpaired T.test	5.B
Trained VS guinea Pig SNR +0	Trained:0.348 Guinea Pig:0.321	Trained:0.016 Guinea Pig:0.012	Trained:304 Guinea Pig:351	0.1483	1.447	653	0.003197	none	Unpaired T.test	5.B
Exposed VS guinea Pig SNR +0	Exposed:0.318 Guinea Pig:0.321	Exposed:0.014 Guinea Pig:0.012	Exposed:266 Guinea Pig:351	0.8603	0.1761	615	5.041e-005	none	Unpaired T.test	5.B
Trained vs Exposed SNR -10	Trained:0.354 Exposed:0.324	Trained:0.017 Exposed:.0.016	Trained:309 Exposed:261	0.1876	1.319	568	0.003055	none	Unpaired T.test	5.B
Trained VS guinea Pig SNR -10	Trained:0.354 Guinea Pig:0.313	Trained:0.017 Guinea Pig:0.011	Trained:309 Guinea Pig:365	0.0339 *	2.126	672	0.006682	none	Unpaired T.test	5.B
Exposed VS guinea Pig SNR -10	Exposed:0.324 Guinea Pig:0.313	Exposed:.0.016 Guinea Pig:0.011	Exposed:261 Guinea Pig:365	0.5718	0.5658	624	0.0005127	none	Unpaired T.test	5.B

Table S7（Original data of Figure 5C）

Variable	Mean value	SEM	n (value)	P**	F (DFn. DFd)	R square	Units	Statistical test	Figure in which data are presented
MI<br>all groups<br>SNR +10	Trained:1.036<br>Exposed:0.843<br>Guinea Pig:1.041	Trained:0.061<br>Exposed:0.056<br>Guinea Pig:0.035	Trained:67<br>Exposed:53<br>Guinea Pig:389	0.0255 *	F (2. 429) = 3.700	0.01696	bit	ANOVA	5.C
MI<br>all groups<br>SNR +0	Trained:0.932<br>Exposed:0.775<br>Guinea Pig:1.052	Trained:0.066<br>Exposed:0.058<br>Guinea Pig:0.038	Trained:58<br>Exposed:46<br>Guinea Pig:389	0.0017 **	F (2. 388) = 6.467	0.03226	bit	ANOVA	5.C
MI<br>all groups<br>SNR -10	Trained:0.813<br>Exposed:0.654<br>Guinea Pig:1.079	Trained:0.058<br>Exposed:0.048<br>Guinea Pig:0.038	Trained:50<br>Exposed:40<br>Guinea Pig:389	<0.0001 ****	F (2. 379) = 17.51	0.08459	bit	ANOVA	5.C

Variable	Mean value	SEM	n (value)	P**	T	DF	η^2 (eta squared)	Units	Statistical test	Figure in which data are presented
Trained vs Exposed<br>SNR+10	Trained:1.036<br>Exposed:0.843	Trained:0.061<br>Exposed:0.056	Trained:67<br>Exposed:53	0.0243 *	2.282	118	0.04227	bit	Unpaired T.test	5.C
Trained VS guinea Pig<br>SNR+10	Trained:1.036<br>Guinea Pig:1.041	Trained:0.061<br>Guinea Pig:0.035	Trained:67<br>Guinea Pig:389	0.94	0.07527	377	1.503e-005	bit	Unpaired T.test	5.C
Exposed VS guinea Pig<br>SNR+10	Exposed:0.843<br>Guinea Pig:1.041	Exposed:0.056<br>Guinea Pig:0.035	Exposed:53<br>Guinea Pig:389	0.0073 **	2.698	363	0.01967	bit	Unpaired T.test	5.C

Variable	Mean value	SEM	n (value)	P**	T	DF	η^2 (eta squared)	Units	Statistical test	Figure in which data are presented
Trained vs Exposed SNR +0	Trained:0.932<br>Exposed:0.775	Trained:0.066<br>Exposed:0.058	Trained:58<br>Exposed:46	0.087	1.728	102	0.02845	bit	Unpaired T.test	5.C
Trained VS guinea Pig SNR +0	Trained:0.932<br>Guinea Pig:1.052	Trained:0.066<br>Guinea Pig:0.038	Trained:58<br>Guinea Pig:389	0.1117	1.595	343	0.007358	bit	Unpaired T.test	5.C
Exposed VS guinea Pig SNR +0	Exposed:0.775<br>Guinea Pig:1.052	Exposed:0.058<br>Guinea Pig:0.038	Exposed:46<br>Guinea Pig:389	0.0007 ***	3.407	331	0.03388	bit	Unpaired T.test	5.C
Trained vs Exposed SNR -10	Trained:0.813<br>Exposed:0.654	Trained:0.058<br>Exposed:0.048	Trained:50<br>Exposed:40	0.0434 *	2.050	88	0.04557	bit	Unpaired T.test	5.C
Trained VS guinea Pig SNR -10	Trained:0.813<br>Guinea Pig:1.079	Trained:0.058<br>Guinea Pig:0.038	Trained:50<br>Guinea Pig:389	0.0007 ***	3.442	340	0.03367	bit	Unpaired T.test	5.C
Exposed VS guinea Pig SNR -10	Exposed:0.654<br>Guinea Pig:1.079	Exposed:0.048<br>Guinea Pig:0.038	Exposed:40<br>Guinea Pig:389	<0.0001 ****	5.043	330	0.07156	bit	Unpaired T.test	5.C
